# Differential effects of phosphate binders on pre-dialysis serum bicarbonate in end-stage kidney disease patients on maintenance haemodialysis

**DOI:** 10.1186/1471-2369-14-205

**Published:** 2013-10-01

**Authors:** Zaw Thet, Aung Ko WIN, Eugenie Pedagogos, Jennifer Beavis, Sandra Crikis, Craig Nelson

**Affiliations:** 1 Department of Nephrology, Western Health, Melbourne, Victoria, Australia; 2 Centre for Molecular, Environmental, Genetic and Analytic Epidemiology, The University of Melbourne, Parkville, Victoria, Australia; 3 Department of Nephrology, Melbourne Health, Melbourne, Victoria, Australia

**Keywords:** Haemodialysis, Metabolic acidosis, Serum bicarbonate, Hyperphosphatemia, Phosphate binders

## Abstract

**Background:**

Phosphate binders’ constituents have alkalotic or acidotic properties and may contribute to acid base balance in haemodialysis patients. This study aimed to investigate the differential effects of phosphate binders on pre-dialysis serum bicarbonate in End Stage Kidney Disease patients on maintenance haemodialysis.

**Methods:**

Stable out-patients having satellite haemodialysis for at least 3 months were retrospectively studied for 18 months, excluding those with other medical causes for metabolic acidosis. Blood results were censored for inpatient episodes, at the time of death, renal transplant or dialysis modality change. Multivariable multilevel mixed-effects linear regression was used and five groups of phosphate binders were compared: Group A(Calcium (Ca) and/or Aluminium (Al) binders); B(Sevelamer hydrochloride (SH) alone); C(lanthanum carbonate (LC) alone); D(SH and Ca/Al), E(LC and Ca/Al).

**Results:**

Of 320 patients, 292 were eligible for analysis with a mean follow-up of 15.54 (standard deviation, SD 3.98) months. Similar mean pre-dialysis serum levels of bicarbonate were observed at all 6 month-interval analyses. At 18^th^ months, observed mean serum bicarbonate levels in mmol/L were Group B: 21.58 (SD 2.82, *P*<0.001), C: 23.29 (SD 2.80, *P*=0.02), D: 21.56 (SD 3.00, *P*<0.001), and E: 21.29 (SD 3.62, *P*=0.92) compared with Group A: 22.98 (SD 2.77). Mean serum bicarbonate was related to total SH dose in mmol/L: 22.34 (SD 2.56) for SH <2.5 g/day, 21.61 (SD 2.62) for SH 2.5-4.8 g/day, 21.04 (SD 3.31) for SH >4.8 g/day compared with 22.85 (SD 2.91) for non-users; P-trend<0.001.

**Conclusions:**

Phosphate binders’ constituents may contribute to/protect against a predisposition to pre-dialysis metabolic acidosis. This may be dose dependant in patients taking Sevelamer Hydrochloride.

## Background

An increased anion gap metabolic acidosis is a characteristic of End Stage Kidney Disease (ESKD). Recognition of the metabolic acidosis in haemodialysis is essential because of the serious complications that can arise. The clinical importance of this is summarized in Table 1[1,2]. According to the Caring for Australians with Renal Impairment (CARI) Guidelines in 2000, pre-dialysis serum bicarbonate in haemodialysis patient should be in the range of 23–24 mmol/L [3]. Contrary to available evidence; a few epidemiologic studies showed that a mild degree of metabolic acidosis was not associated with increased risk of morbidity and mortality [4,5]. In 2009, the British Renal Association recommended that pre-dialysis serum bicarbonate concentrations, measured with minimum delay after venepuncture, should be between 18 and 24 mmol/L [6].

**Table 1:** Potential adverse effects of metabolic acidosis in patients with chronic kidney disease

**Effect**	**Comment**
Muscle wasting	Seen with even mild metabolic acidosis (important factor in causing muscle wasting in patients with CKD)
Reduced albumin synthesis	Acidosis is one of many factors contributing to hypoalbuminemia in patients with CKD
Bone disease	Acidosis contributes to the genesis of bone disease by diverse mechanisms; contributory rather than primary mechanism in producing bone disease
	Direct effects
	• Physicochemical dissolution of bone • Decreased function of osteoblast
	• Increased function of osteoclast
	Indirect effects
	• Increased release of parathyroid hormone
	• Increased number of parathyroid hormone receptors
	• Increased binding of parathyroid hormone to its receptor
	• Decreased activity of 1-a hydroxylase
Impaired insulin sensitivity	Effect unclear given the impact of changes in insulin metabolism with renal failure; could induce metabolic changes similar to those seen in syndrome X*
B2-Microglobulin accumulation	Found with studies of acetate *v* bicarbonate dialysis in dialysis patients; no studies of patients with CKD not on dialysis therapy
Exacerbation of renal failure	Data for and against role of acidosis in progression of renal failure
Impaired thyroid metabolism	May contribute to abnormalities in basal metabolic rate
Stunted growth in children	Reversed in part by correction of acidosis
Cardiac disease	Role in the development of cardiac disease is theoretical, not proven
Increased inflammation	Conflicting evidence for and against the role of acidosis in dialysis patients

Syndrome X^*^ is characterized by dislipidemia, hyperinsulinemia, hypertension, abdominal obesity, glucose intolerance and renal resistance.

From Kraut JA et al. [1]: Metabolic Acidosis of CKD: Diagnosis, Clinical Characteristics and Treatment. AJKD 2005;45(6):978–93.

Hyperphosphataemia, a frequent and serious complication of ESKD, partially contributes to metabolic acidosis which can be temporarily corrected by haemodialysis. Phosphate binders presently available in Australia include aluminium (Al) binders, calcium (Ca) binders and non-calcium and non-aluminium binders such as sevelamer hydrochloride (SH) and lanthanum carbonate (LC). Phosphate binders’ constituents have alkalotic or acidotic properties and may contribute to acid base balance in haemodialysis patients. Hyperphosphatemia itself and phosphate binders may influence the acid–base status of the patients. This study aimed to investigate the differential effects of phosphate binders on pre-dialysis serum bicarbonate in ESKD patients on maintenance haemodialysis when comparing patients taking different binders.

Aluminium (Al) binders contain an alkali that might partially compensate metabolic acidosis in ESKD patients. Aluminium phosphate binders are generally avoided because of concerns of end-organ damage. As a result, aluminium binders have largely been replaced by the calcium based binders. Calcium carbonate as a phosphate binder delivers an alkali and may raise serum bicarbonate. Similarly the acetate in calcium acetate when used as a phosphate binder is metabolized to bicarbonate in the liver. The efficacy of calcium phosphate binders is offset by the potential side effects associated with increased calcium absorption. Sevelamer hydrochloride is a non-aluminum and non-calcium phosphate binder which has proven to be effective in lowering phosphate without raising calcium levels and causing harmful effects on renal bone disease [7,8]. SH acts like an ion-exchange resin that releases chloride in exchange of phosphate ions. The chloride thus released is buffered by bicarbonate, contributing to metabolic acidosis [9-14]. Lanthanum carbonate is a non- aluminium, non-calcium, phosphate binder that is effective and well tolerated. Lanthanum carbonate contains an alkali and it is expected to raise bicarbonate and act as a buffer. However, there is a concern regarding potential lanthanum accumulation in the tissues over time and possible long term side-effects. Despite concerns about long-term effects of lanthanum, long term follow up for 6 years has not supported these concerns [15].

Adequate, safe control of phosphate and calcium levels with phosphate binders is therefore difficult and multiple agents may be required. In Australia, first line treatment for hyerphosphataemia remains calcium based phosphate binders in patients with low risk for vascular calcification because of Pharmaceutical Benefit Scheme (PBS) restrictions on using non-calcium and non-aluminum binders in CKD and denovo in haemodilysis. Second line treatment such as SH or LC is indicated with or without combination of first line binders only in patients with high risk of vascular calcification and/or hyperphosphatemia that is not controlled by first line phosphate binders.

There is a tendency in practice to combine phosphate binders in an attempt to improve efficacy or to minimize side effects of binders. Although there are cross-over or head to head studies comparing efficacy of phosphate binders and suggesting increased risk of metabolic acidosis in patients treated with SH, there is no study to investigate long term individual and combined effects of phosphate binders on acid base balance in haemodialysis patients. To our knowledge, our study will be the first of its kind in Australian setting and it is close to a real life setting.

## Methods

### Patients

This retrospective study included 292 stable Melbourne Health and Western Health out-patients who received satellite haemodialysis for a period of at least three months. Both Western & Melbourne Health units practiced the same audit policy in order to achieve CARI Guidelines’ target of pre-dialysis bicarbonate of 23–24 mmol/L. It was a standard practice to supplement alkai by increasing dialysate bicarbonate concentration to 40 mmol/L from 35 mmol/L at monthly blood audit in both units when serum bicarbonate fell below 18 mmol/L. In order to avoid post-dialysis alkalemia, dialysate bicarbonate concentrations were modified back to 35 mmol/L when pre-dialysis serum bicarbonate was ≥24 mmol/L. The monthly blood tests were audited by these rules in both centres by a single Nephrologist in the respective centres.

This study was approved by Melbourne Health and Western Health Ethics committees. Patients with other medical causes for metabolic acidosis were excluded. Exclusion criteria were alcohol abuse, recurrent hypoglycemia, decompensated organ failure, cancer with active treatment or life expectancy less than a year, recurrent diabetic ketoacidosis, severe gastromotility disorders, pancreatic or biliary fistula, uretosigmoidostomy, long term nephrostomy tube and renal tubular acidosis. These audited patients were stable out-patients having dietary advice routinely in out-patient clinics and dialysis three times per week. Monthly patient information was recorded from December 2008 to June 2010. All blood samples were analyzed without delay by Melbourne Health Pathology by standard laboratory methods. Blood results were censored for inpatient episodes, at the time of death, renal transplant or dialysis modality change.

### Statistical analysis

Long term individual and combined effects of phosphate binders on pre-dialysis serum bicarbonate, phosphate, potassium and corrected calcium were compared for five groups: Group A (Calcium (Ca) and/or Aluminium (Al) binders); B (Sevelamer hydrochloride (SH) alone); C (lanthanum carbonate (LC) alone); D (SH and Ca/Al), E (LC and Ca/Al). Pre-dialysis serum bicarbonate, phosphate, potassium and corrected calcium were also compared for three dosing groups of SH: low dose (SH <2.5 g/day), medium dose (SH 2.5-4.8 g/day) and high dose (SH >4.8 g/day). Allowing both random and fixed effects of medication along the study period, multilevel mixed-effects linear regression was used to estimate mean differences and 95% confidence intervals (CIs) at three different time points by six months intervals (June 2009, December 2009 and June 2010) after adjusting for sex, age and diabetes. All reported statistical tests were two-sided and *P*<0.05 was considered statistically significant. All statistical analyses were done using Stata 11.0 [16].

## Results

Of 320 patients, 292 (63% male) were eligible for analysis with a mean follow-up of 15.54 (standard deviation, SD 3.98) months. Baseline characteristics of these patients are shown in Table 2. The majority of patients was Caucasian (55%) and had been on dialysis for, on average, 2 years (mean 49.22 months; SD 39.65). Mean urea reduction ratio (URR) was 72.76% (SD 6.96). Diabetes mellitus (40%) was the most common aetiology of ESKD in this study.

**Table 2:** Baseline characteristics of patients in this study

	**Total no (%) (n=292)**	**Group A (n=108)**	**Group B (n=80)**	**Group C (n=24)**	**Group D (n=60)**	**Group E (n=20)**
Sex						
Male	184 (63)	65	49	14	41	15
Female	108 (37)	43	31	10	19	5
Race						
Caucasian	162 (55)	57	48	20	30	7
Asian	38 (13)	15	9	0	14	0
African	6 (2)	2	1	0	2	1
Australian Aborigines	1 (0.5)	1	0	0	0	0
Pacific Islander	7 (2.5)	4	2	0	1	0
Middle Eastern	14 (5)	6	4	0	2	2
Other	11 (4)	5	2	0	4	0
Unknown	53 (18)	18	14	4	7	10
Aetiology						
Polycystic kidney disease	12 (4)	1	5	1	3	2
Diabetes type II	70 (24)	23	23	3	18	3
Diabetes type I	46 (16)	21	5	5	10	5
Focal Segmental Glomerular Sclerosis	31 (11)	12	4	4	7	4
Glomerular Nephritis	21 (7)	5	7	4	4	1
Hypertension	4 (1)	1	1	1	1	0
Idiopathic	2 (1)	1	0	0	1	0
Ig A disease	24 (8)	10	5	1	6	2
Reflux nephropathy	14 (5)	6	5	1	2	0
Other	17 (6)	7	8	1	1	0
Renal Calculus	2 (1)	1	1	0	0	0
Renovascular disease	10 (3)	4	4	1	1	0
Unknown	39 (13)	16	12	2	6	3
Diabetes						
Yes	138 (47)	51	37	11	29	10
No	154 (53)	57	43	13	31	10
Ischaemic heart disease						
Yes	117 (40)	50	24	7	26	10
No	148 (51)	48	48	15	30	7
Unknown	27 (9)	10	8	2	4	3
Parathyroidectomy						
Yes	17 (6)	6	2	1	7	1
No	275 (94)	102	78	23	53	19
	**Mean (SD)**					
Age (year)	65.56 (14.40)	67.51 (13.90)	66.33 (14.82)	66.29 (16.31)	62.06 (13.39)	61.83 (15.50)
Haemodialysis period (month)	49.22 (39.65)	46.52 (40.58)	47.86 (39.74)	75.25 (40.79)	49.06 (35.56)	38.10 (15.56)
Urea Reduction Ratio	72.76 (6.96)	71.76 (8.08)	73.93 (6.20)	75.30 (6.51)	71.99 (6.07)	73.30 (5.21)
Follow-up duration (month)	15.54 (3.98)	15.22 (4.22)	16.12 (3.15)	16.02 (3.29)	16.39 (3.37)	15.47 (4.14)

SD=standard deviation.

Group A (Calcium and/or Aluminum binders).

Group B (Sevelamer hydrochloride alone).

Group C (Lanthanum carbonate alone).

Group D (Sevelamer hydrochloride and Calcium and/or Aluminum binders).

Group E (Lanthanum carbonate and Calcium and/or Aluminum binders).

Table 3 shows phosphate binder usage at different time points of 6 month-interval analysis: Group A (Ca/Al) approximately 34%-37%, Group B (SH) 27%-29%, Group C (LC) 8%-9%, Group D (SH plus Ca/Al) 21%-23% and Group E (LC plus Ca/Al) 7%-8%. Because of Australian PBS restrictions at the time, the number of patients using lanthanum carbonate was small. Therefore, results of group C (LC) and group E (LC plus Ca/AL) should be interpreted with caution. Majority (approximately 90-95%) remained on the same binders at each of the 6 month study period. Both Melbourne and Western Health units phased out an oral bicarbonate supplementation method. During our study period, auditors identified only 2% of the cohort who remained on oral sodium bicarbonate and terminated the treatment completely. Patients who required an increased dose of dialysate bicarbonate were summarized in Table 4. Overall, compared to Group (A) Ca/Al, Group B (SH) and Group D (SH plus Ca/Al) received more alkali supplementation by increasing dose of dialysate bicarbonate.

**Table 3:** **Number of patients** (%) **at each category of phosphate binders usage at 6 month interval of analysis**

**Phosphate binders**	**June 2009 (n=292)**	**December 2009 (n=260)**	**June 2010 (n=237)**
Group A (Ca & Al)	108 (37)	83 (32)	81 (34)
Group B (SH)	80 (27)	76 (29)	68 (29)
Group C (LC)	24 (8)	22 (8)	21 (9)
Group D (SH & Ca/Al)	60 (21)	59 (23)	50 (21)
Group E (LC & Ca/Al)	20 (7)	20 (8)	17 (7)

Group A (Calcium and/or Aluminum binders).

Group B (Sevelamer hydrochloride alone).

Group C (Lanthanum carbonate alone).

Group D (Sevelamer hydrochloride and Calcium and/or Aluminum binders).

Group E (Lanthanum carbonate and Calcium and/or Aluminum binders).

**Table 4:** **Number of patients** (%) **who required increased dose of dialysate bicarbonate**

	**Before June 2009 (N=51/292)**	**Between July 2009 and December 2009 (N=37/260)**	**Between January 2010 and June 2010 (N=21/237)**
Group A (Ca & Al)	10/108	5/83	6/81
Group B (SH)	21/80	17/76	6/68
Group C (LC)	0/24	0/22	1/21
Group D (SH & Ca/Al)	19/60	13/59	5/50
Group E (LC & Ca/Al)	1/20	2/20	3/17
**P Value (B&D cf. A)**	**<0.001**	**<0.004**	**0.67**

Group A (Calcium and/or Aluminum binders).

Group B (Sevelamer hydrochloride alone).

Group C (Lanthanum carbonate alone).

Group D (Sevelamer hydrochloride and Calcium and/or Aluminum binders).

Group E (Lanthanum carbonate and Calcium and/or Aluminum binders).

**Figure 1:**
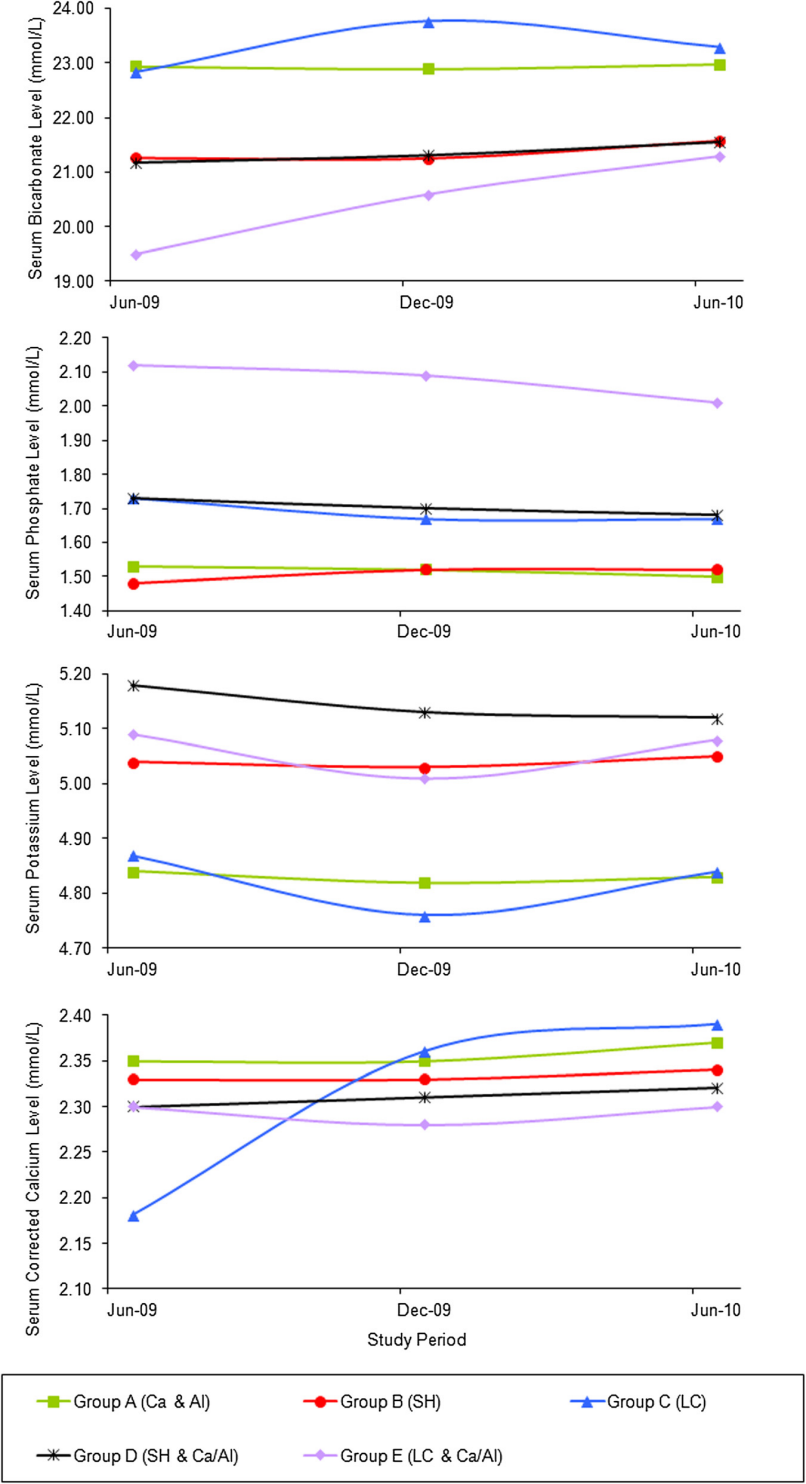
**Pre-dialysis mean serum level of bicarbonate, phosphate, potassium and corrected calcium different combinations of phosphate binders.**

Pre-dialysis observed mean levels and estimated mean differences of serum bicarbonate, phosphate, potassium, and corrected calcium for all five phosphate binder groups were shown in Table 5. Figure 1 reveals the time course changes in pre-dialysis parameters: observed mean levels of serum bicarbonate, phosphate, potassium and corrected calcium. When mean serum bicarbonate levels were compared with Group A (Ca/Al), serum bicarbonate levels were significantly lower in group B (SH) and group D (SH plus Ca/Al) (*P*<0.001) at all points of 6 month interval analysis. Pre-dialysis serum potassium was marginally elevated in Group B (SH) and Group D (SH plus Ca/Al) (*P*<0.001) compared with Group A (Ca/Al).

**Table 5:** **Pre**-**dialysis mean serum level of bicarbonate**, **phosphate**, **potassium and corrected calcium for different combinations of phosphate binders**

	**June 2009**			**December 2009**			**June 2010**		
	**Mean (SD)**^**#**^	Mean difference (95% CI)^*^	***P***	**Mean (SD)**^**#**^	Mean difference (95% CI)^*^	**P**	**Mean (SD)**^**#**^	Mean difference (95% CI)^*^	**P**
	**Bicarbonate**								
Group A (Ca & Al)	22.94 (2.83)	ref		22.89 (2.76)	ref		22.98 (2.77)	ref	
Group B (SH)	21.26 (2.72)	-1.56 (-2.09 to -1.02)	<0.001	21.25 (2.83)	-1.18 (-1.59 to -0.78)	<0.001	21.58 (2.82)	-0.95 (-1.29 to -0.60)	<0.001
Group C (LC)	22.83 (1.17)	-0.01 (-2.76 to 2.74)	0.98	23.76 (2.57)	0.74 (0.05 to 1.43)	0.04	23.29 (2.80)	0.58 (0.10 to 1.05)	0.02
Group D (SH & Ca/Al)	21.17 (3.00)	-1.30 (-1.83 to -0.77)	<0.001	21.31 (3.00)	-0.69 (-1.10 to -0.29)	<0.001	21.56 (3.00)	-0.64 (-1.00 to -0.28)	<0.001
Group E (LC & Ca/Al)	19.50 (4.45)	-1.40 (-2.79 to 0.00)	0.05	20.59 (3.59)	-0.44 (-1.09 to 0.22)	0.19	21.29 (3.62)	-0.03 (-0.54 to 0.49)	0.92
	**Phosphate**^**^**^								
Group A (Ca & Al)	1.53 (0.51)	ref		1.52 (0.50)	ref		1.50 (0.49)	ref	
Group B (SH)	1.48 (0.41)	-0.01 (-0.10 to 0.08)	0.83	1.52 (0.44)	0.03 (-0.04 to 0.10)	0.35	1.52 (0.45)	-0.01 (-0.07 to 0.05)	0.70
Group C (LC)	1.73 (0.63)	0.32 (-0.12 to 0.77)	0.16	1.67 (0.54)	0.08 (-0.04 to 0.19)	0.18	1.67 (0.53)	0.04 (-0.03 to 0.12)	0.27
Group D (SH & Ca/Al)	1.73 (0.57)	0.08 (-0.02 to 0.17)	0.11	1.70 (0.54)	0.02 (-0.05 to 0.09)	0.65	1.68 (0.55)	0.00 (-0.06 to 0.06)	0.97
Group E (LC & Ca/Al)	2.12 (0.58)	0.29 (0.05 to 0.52)	0.02	2.09 (0.74)	0.16 (0.06 to 0.27)	0.003	2.01 (0.77)	0.06 (-0.02 to 0.15)	0.14
	**Potassium**								
Group A (Ca & Al)	4.84 (0.70)	ref		4.82 (0.69)	ref		4.83 (0.70)	ref	
Group B (SH)	5.04 (0.71)	0.14 (0.01 to 0.27)	0.04	5.03 (0.72)	0.17 (0.07 to 0.27)	<0.001	5.05 (0.70)	0.16 (0.08 to 0.24)	<0.001
Group C (LC)	4.87 (0.36)	0.21 (-0.45 to 0.87)	0.54	4.76 (0.74)	-0.02 (-0.18 to 0.14)	0.84	4.84 (0.81)	0.05 (-0.06 to 0.16)	0.38
Group D (SH & Ca/Al)	5.18 (0.72)	0.20 (0.06 to 0.33)	<0.001	5.13 (0.69)	0.14 (0.04 to 0.23)	0.01	5.12 (0.68)	0.15 (0.06 to 0.23)	<0.001
Group E (LC & Ca/Al)	5.09 (0.62)	0.18 (-0.16 to 0.51)	0.30	5.01 (0.79)	-0.04 (-0.19 to 0.12)	0.63	5.08 (0.77)	0.03 (-0.10 to 0.15)	0.68
	**Corrected Calcium**^**^**^								
Group A (Ca & Al)	2.35 (0.19)	ref		2.35 (0.18)	ref		2.37 (0.18)	ref	
Group B (SH)	2.33 (0.19)	-0.03 (-0.06 to 0.01)	0.14	2.33 (0.19)	-0.03 (-0.06 to -0.01)	0.01	2.34 (0.19)	-0.04 (-0.06 to -0.02)	<0.001
Group C (LC)	2.18 (0.15)	-0.12 (-0.30 to 0.05)	1.17	2.36 (0.18)	-0.01 (-0.05 to 0.03)	0.73	2.39 (0.18)	0.01 (-0.02 to 0.04)	0.60
Group D (SH & Ca/Al)	2.30 (0.20)	-0.05 (-0.09 to -0.02)	0.004	2.31 (0.19)	-0.04 (-0.06 to -0.01)	0.003	2.32 (0.19)	-0.04 (-0.06 to -0.02)	<0.001
Group E (LC & Ca/Al)	2.30 (0.23)	-0.03 (-0.12 to 0.06)	0.56	2.28 (0.22)	-0.01 (-0.05 to 0.03)	0.50	2.30 (0.21)	-0.02 (-0.05 to 0.01)	0.29

Group A (Calcium and/or Aluminium binders).

Group B (Sevelamer hydrochloride alone).

Group C (Lanthanum carbonate alone).

Group D (Sevelamer hydrochloride and Calcium and/or Aluminium binders).

Group E (Lanthanum carbonate and Calcium and/or Aluminium binders).

^#^Observed mean level in mmol/L (standard deviation).

*Estimated mean difference in mmol/L (95% confidence interval) after adjusted for sex, age and diabetes.

^^^for those patients who had no parathyroidectomy.

**Figure 2:**
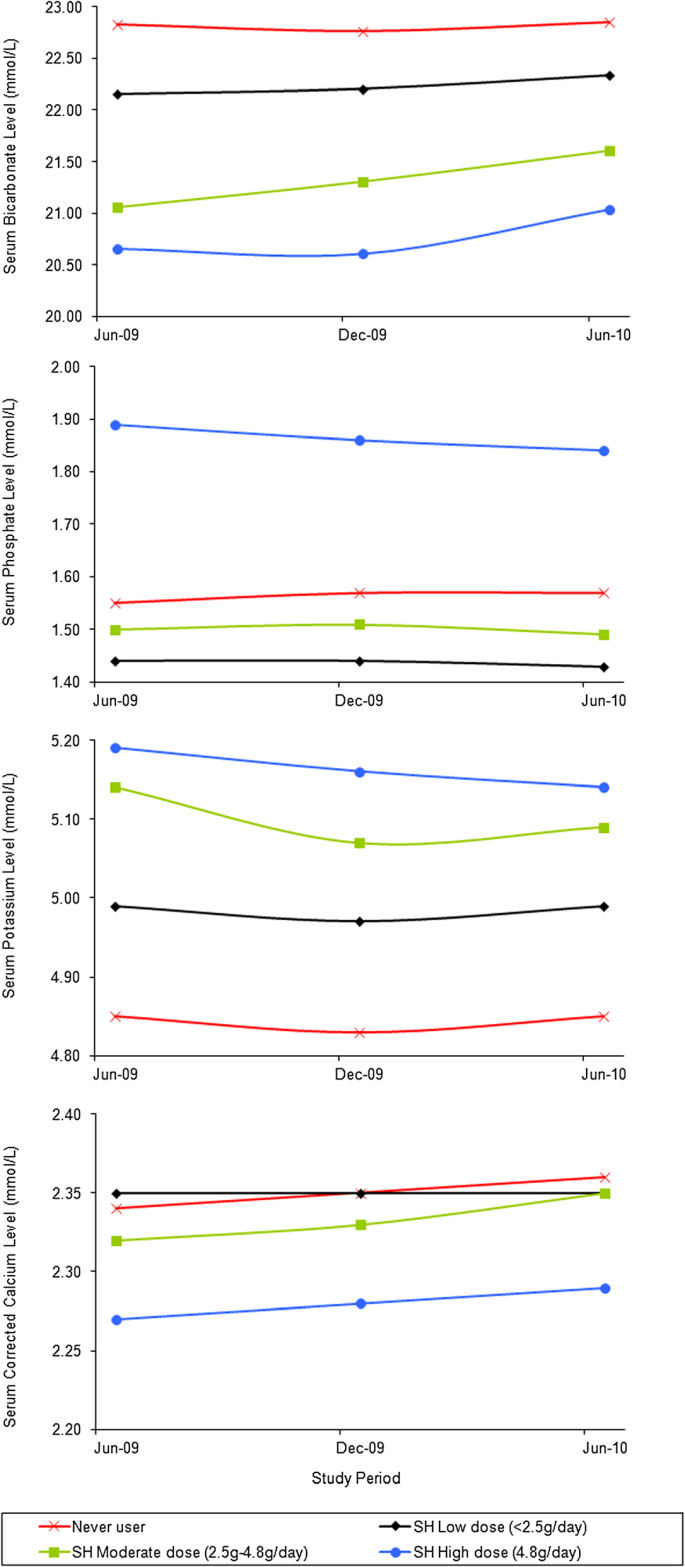
**Pre**-**dialysis mean serum bicarbonate**, **phosphate**, **potassium and corrected calcium for separate doses of sevelamer hydrochloride.**

In sub-group analysis, patients taking SH (alone or with other binders) were compared in three dosing groups (Table 6 and Figure 2). A significant correlation between dosages of SH and pre-dialysis serum bicarbonate, potassium and corrected calcium levels was observed over the whole study period. The higher the dose of SH given in ESKD patients on maintenance haemodialysis, the lower the mean serum bicarbonate level and the higher the mean serum potassium level (*P*-trend <0.001). There were no major difference in mean serum pre-dialysis phosphate at the end of study (*P*-trend =0.53). Mean serum corrected calcium (p <0.001) was marginally decreased in patients treated with SH (SH alone and/or conventional binders) when compared to non-SH users at all points of 6 month-interval sub-group analysis.

**Table 6:** **Pre**-**dialysis mean serum bicarbonate**, **phosphate**, **potassium and corrected calcium for separate doses of sevelamer hydrochloride**

	**June 2009**			**December 2009**			**June 2010**		
**Sevelamer hydrochloride**	**Mean (SD)**^**#**^	Mean difference (95% CI)*	***P*-trend**	**Mean (SD)**^**#**^	Mean difference (95% CI)*	***P*-trend**	**Mean (SD)**^**#**^	Mean difference (95% CI)*	***P*-trend**
	**Bicarbonate**									
Non SH user*	22.83 (2.93)	ref		22.77 (2.88)	ref		22.85 (2.91)	ref		
Low dose	22.16 (2.53)	-0.56 (-1.16 to 0.04)		22.21 (2.53)	-0.16 (-0.63 to 0.32)		22.34 (2.56)	-0.22 (-0.63 to 0.20)		
Moderate dose	21.06 (2.68)	-1.66 (-2.18 to -1.13)		21.31 (2.62)	-0.99 (-1.39 to -0.59)		21.61 (2.62)	-0.94 (-1.27 to -0.61)		
High dose	20.66 (3.14)	-1.89 (-2.51 to -1.26)	<0.001	20.61 (3.30)	-1.60 (-2.03 to -1.16)	<0.001	21.04 (3.31)	-1.27 (-1.63 to -0.91)	<0.001	
	**Phosphate**^**^**^									
Non SH user*	1.55 (0.52)	ref		1.57 (0.54)	ref		1.57 (0.55)	ref		
Low dose	1.44 (0.43)	-0.07 (-0.17 to 0.03)		1.44 (0.40)	-0.09 (-0.17 to -0.01)		1.43 (0.42)	-0.06 (-0.13 to 0.01)		
Moderate dose	1.50 (0.44)	0.03 (-0.06 to 0.12)		1.51 (0.45)	-0.01 (-0.08 to 0.06)		1.49 (0.46)	-0.05 (-0.10 to 0.01)		
High dose	1.89 (0.54)	0.14 (0.03 to 0.26)	0.03	1.86 (0.53)	0.08 (0.01 to 0.16)	0.07	1.84 (0.53)	0.04 (-0.02 to 0.10)	0.53	
	**Potassium**									
Non SH user*	4.85 (0.70)	ref		4.83 (0.70)	ref		4.85 (0.72)	ref		
Low dose	4.99 (0.68)	0.07 (-0.07 to 0.22)		4.97 (0.67)	0.09 (-0.03 to 0.20)		4.99 (0.65)	0.07 (-0.03 to 0.17)		
Moderate dose	5.14 (0.72)	0.20 (0.07 to 0.33)		5.07 (0.70)	0.16 (0.07 to 0.26)		5.09 (0.69)	0.15 (0.07 to 0.23)		
High dose	5.19 (0.72)	0.21 (0.05 to 0.37)	<0.001	5.16 (0.72)	0.21 (0.11 to 0.32)	<0.001	5.14 (0.70)	0.17 (0.09 to 0.26)	<0.001	
	**Corrected Calcium**^**^**^									
Non SH user*	2.34 (0.19)	ref		2.35 (0.19)	ref		2.36 (0.19)	ref		
Low dose	2.35 (0.18)	-0.04 (-0.08 to 0.00)		2.35 (0.18)	-0.03 (-0.06 to 0.00)		2.35 (0.18)	-0.04 (-0.06 to -0.01)		
Moderate dose	2.32 (0.20)	-0.03 (-0.07 to 0.00)		2.33 (0.19)	-0.03 (-0.06 to -0.01)		2.35 (0.18)	-0.04 (-0.06 to -0.02)		
High dose	2.27 (0.19)	-0.05 (-0.10 to -0.01)	0.02	2.28 (0.18)	-0.05 (-0.08 to -0.02)	<0.001	2.29 (0.18)	-0.04 (-0.07 to -0.02)	<0.001	

SH Low Dose (SH <2.5 g/day) – 1 to 3 tabs per day.

SH Medium Dose (SH 2.5-4.8 g/day) – 4 to 6 tabs per day.

SH High Dose (SH >4.8 g/day) – 7 to 9 tabs per day.

^#^Observed mean level in mmol/L (standard deviation).

*Estimated mean difference in mmol/L (95% confidence interval) after adjusted for sex, age and diabetes.

^^^for those patients who had no parathyroidectomy.

## Discussion

In our study, the control of serum phosphate by SH in ESKD patients might occur at the expense of lowering serum bicarbonate. The higher the dose of SH given in ESKD patients, the lower the mean serum bicarbonate level. Hyperkalemia may be explained by aggravation of metabolic acid by SH. Oka et al., reported that the levels of pre-dialysis serum bicarbonate significantly and negatively correlated with the daily SH dosage [12]. Metabolic acidosis in hemodialysis patients may be associated with other causes related to dialysis and its prescription. In our study, mean URR suggested that patients were adequately dialyzed with no difference between the study groups. In our study, other important dialysis/patients related factors: for example- interdialytic fluid gain, administration of alkali, excessive protein intake, hypotension during dialysis and chronic hyperparathyroidism were not taken into account in interpretation of metabolic acidosis. In 2009, the British Renal Association (BRA) guidelines recommended lower level of pre-dialysis serum bicarbonate levels than the targets of earlier local (CARI, 2000) and international (The National Kidney Foundation Disease Outcomes Quality Initiative KDOQI, 2003) guidelines based on the evidence of epidemiologic studies [3,6,17]. As epidemiologic analysis can identify only an association of metabolic acidosis and patients’ mortality/morbidity, causal relationships need to be demonstrated by clinical trials. Hence, the results of epidemiologic data analysis should be interpreted with caution.

SH, LC and Ca/Al phosphate binders are very effective in controlling serum phosphate and calcium levels [7,8]. The recommended Caring for Australians with Renal Impairment (CARI) targets for pre-dialysis serum phosphate and corrected calcium were 2.1-2.4 mmol/L and 0.8-1.6 mmol/L respectively. Mean pre-dialysis serum corrected calcium levels were within the CARI targets. In our study, mean serum corrected calcium (*P*<0.001) was marginally decreased in patients treated with SH when compared to conventional binder users. A study of ten-year experience with sevelamer and calcium binders suggested that sevelamer might improve vascular and bone health and perhaps, mortality in haemodialysis patients [8]. Our serum phosphate levels were above the targets in all five groups of patients. A number of other factors which were not reviewed in this study may have contributed to the poor achievement of serum phosphate target levels, including poor patient insight into the consequences of hyperphosphatemia, variable dietary management, and poor adherence with binders, Hyperphosphataemia itself partially contributes to metabolic acidosis. As mean phosphate levels did not differ significantly between the groups at the end of our study, differences in mean serum bicarbonate among the groups could not be explained by hyperphosphatemia.

Several investigators have studied metabolic acidosis treatment in ESKD patients on maintenance haemodialysis. Alkali has usually been provided either by increasing dialysate bicarbonate concentration, oral bicarbonate administration, or a combination of both [18-24]. Oral administration of sodium bicarbonate may result in volume overload and possibly diminish the effectiveness of sevelamer, as the alkali may compete with phosphate for binding to the resin. Higher bicarbonate administration through dialysis does not result in thirst increase, fluid overload, or significant interdialytic weight gain variation, which are common undesirable effects observed during the oral administration of alkali salts. Sonikian et al. study [13] established that sevelamer-induced acidosis could be successfully managed on the long-term by increasing dialysate bicarbonate concentration. Sevelamer carbonate (SC) is as good as sevelamer hydrochloride (SH) in terms of hyperphosphatemia control with a better outcome in serum bicarbonate balance [25]. However, SC is not available in Australia. Alternatively, switching from SH to lanthanum carbonate (LC) may improve metabolic acidosis and hyeprkalemia [26-28]. In our units (both Melbourne Health & Western Health), it was a standard practice to supplement bicarbonate at monthly blood audit when serum bicarbonate fell below 18. Given the results, the use of these supplements either bicarbonate bath 40 or oral supplements would only bias toward a negative effect in the study for subjects taking sevelamer hydrochloride. There are no data on the most appropriate method for achieving the target bicarbonate. Complete correction of pre-dialysis metabolic acidosis in haemodialysis patients may lead to post-dialysis metabolic alkalosis and consequently hypoventilation, phosphate transfer into cells and a higher risk of soft tissue and vascular calcification. Therefore, further studies are needed to identify the most appropriate target of serum bicarbonate in Haemodialysis patients and the most appropriate method for achieving the target bicarbonate.

## Conclusion

There is a tendency in practice to combine phosphate binders in an attempt to improve efficacy or to minimize side effects of binders. Phosphate binders’ constituents have alkalotic or acidotic properties and may contribute to/protect against a predisposition to metabolic acidosis pre-dialysis. This may be dose dependant in patients taking SH. Clinical implications of phosphate binders related metabolic acidosis need to be determined in prospective studies.

## Competing interest

The authors declare that they have no competing interests.

## Authors’ contribution

ZT collected the data, drafted the article, reviewed the literature and revised it critically. AW did statistical interpretation and provided valuable inputs in the draft. EP provided valuable inputs in study design, data collection and literature review. JB collected the data of Melbourne Health. SC provided valuable inputs in data collection and literature review. CN provided valuable inputs in study design, data collection, literature review, revision of the draft. All authors read and approved the manuscript and met the criteria for authorship.

## Pre-publication history

The pre-publication history for this paper can be accessed here:

http://www.biomedcentral.com/1471-2369/14/205/prepub

## References

[B1] Kraut JA Kurtz I Metabolic acidosis of CKD: diagnosis, clinical characteristics, and treatment Am J Kidney Dis 2005 45 6 978 993 10.1053/j.ajkd.2005.03.003 15957126

[B2] Mehrotra RD KOPPLEJ Wolfson M Metabolic acidosis in maintenance dialysis patients: clinical considerations Kidney Int 2003 64 S88 S13 S26 10.1046/j.1523-1755.2003.08802.x 14870874

[B3] CARI Caring for Austrlisians with Renal Impairment Dialysis Guidlines:Biochemical and Haematological targets (published 2000 March) 2000 Available from http://www.cari.org.au/

[B4] Bommer J Locatelli F Satayathum S Keen ML Goodkin DA Saito A Akiba T Port FK Young EW Association of predialysis serum bicarbonate levels with risk of mortality and hospitalization in the Dialysis Outcomes and Practice Patterns Study (DOPPS) Am J Kidney Dis 2004 44 4 661 671 15384017

[B5] Wu D Kilpatrick R Dadres S McAllister CJ Kopple JD Kalantarâ€Zadeh K Association between serum bicarbonate and death in hemodialysis patients: Is it better to be acidotic or alkalotic? Hemodial Int 2005 9 1 87 87 10.2215/CJN.00010505 17699193

[B6] The Renal Association Laboratory and clinical indices of dialysis adequacy other than dialysis dose 2009 Guidlines 6.3 - HD: Pre-Dialysis serum bicarbonate concentrations (Updated 2009 December). Available from http://www.renal.org/home.aspx

[B7] Sprague SM A comparative review of the efficacy and safety of established phosphate binders: calcium, sevelamer, and lanthanum carbonate* Curr Med Res OpinÂ® 2007 23 12 3167 3175 10.1185/030079907X242719 17991307

[B8] Raggi P Vukicevic S MoysÃ©s RM Wesseling K Spiegel DM Ten-year experience with sevelamer and calcium salts as phosphate binders Clin J Am Soc Nephrol 2010 5 31 40 10.2215/CJN.05880809 20089501

[B9] Brezina B Qunibi WY Nolan CR Acid loading during treatment with sevelamer hydrochloride: mechanisms and clinical implications Kidney Int 2004 66 S39 S45 10.1111/j.1523-1755.2004.09007.x 15296506

[B10] De Santo NG Frangiosa A Anastasio P Marino A Correale G Perna A Di Stazio E Stellato D Santoro D Di Meglio E Sevelamer worsens metabolic acidosis in hemodialysis patients Journal of nephrology 2006 19 S108 16736432

[B11] Sonikian MA Pani IT Iliopoulos AN Koutala KG Marioli SI Vlassopoulos DA Metabolic acidosis aggravation and hyperkaliemia in hemodialysis patients treated by sevelamer hydrochloride Renal failure 2005 27 2 143 147 15807177

[B12] Oka Y Miyazaki M Takatsu S Kunitomo K-i Uno F Maruyama M Matsuda H Sevelamer Hydrochloride Exacerbates Metabolic Acidosis in Hemodialysis Patients, Depending on the Dosage Ther Apher Dial 2007 11 2 107 113 10.1111/j.1744-9987.2007.00432.x 17381531

[B13] Sonikian M Metaxaki P Iliopoulos A Marioli S Vlassopoulos D Long-term management of sevelamer hydrochloride-induced metabolic acidosis aggravation and hyperkalemia in hemodialysis patients Renal failure 2006 28 5 411 418 10.1080/08860220600599092 16825091

[B14] Savica V Santoro D Monardo P Mallamace A Bellinghieri G Sevelamer carbonate in the treatment of hyperphosphatemia in patients with chronic kidney disease on hemodialysis Ther Clin Risk Manag 2008 4 4 821 19209264 10.2147/tcrm.s3075 PMC2621379

[B15] Hutchison AJ Barnett ME Krause R Kwan JTC Siami GA Long-term efficacy and safety profile of lanthanum carbonate: results for up to 6 years of treatment Nephron Clin Pract 2008 110 1 c15 c23 10.1159/000149239 18667837 PMC2790759

[B16] StataCorp Stata Statistical Software: Release 11. College Station, TX: StataCorp LP 2009

[B17] National Kidney Foundation K/DOQI clinical practice guidelines for bone metabolism and disease in chronic kidney disease Am J Kidney Dis 2003 42 3 1 201 14520607

[B18] RAULT R Optimal dialysate bicarbonate during hemodialysis ASAIO J 1991 37 3 M372 1751193

[B19] Zucchelli P Santoro A Correction of acid–base balance by dialysis Kidney Int Suppl 1993 41 S179 8320915

[B20] Williams A Dittmer I McArley A Clarke J High bicarbonate dialysate in haemodialysis patients: effects on acidosis and nutritional status Nephrol Dial Transplant 1997 12 12 2633 10.1093/ndt/12.12.2633 9430864

[B21] Agroyannis B Fourtounas C Tzanatos H Dalamangas A Vlahakos D Relationship Between Interdialytic Weight Gain and Acidâ€ Base Status in Hemodialysis by Bicarbonate Artificial organs 2002 26 4 385 387 10.1046/j.1525-1594.2002.06883.x 11952511

[B22] Oettinger CW Oliver JC Normalization of uremic acidosis in hemodialysis patients with a high bicarbonate dialysate J Am Soc Nephrol 1993 3 11 1804 1807 8329675 10.1681/ASN.V3111804

[B23] Roderick PJ Willis NS Blakeley S Jones C Tomson C Correction of chronic metabolic acidosis for chronic kidney disease patients Cochrane Database of Systematic Reviews 2007 Issue 1. Art. No.: CD001890. DOI: 10.1002/14651858.CD001890.pub3 10.1002/14651858.CD001890.pub3 PMC7045985 17253467

[B24] Noh U Yi JH Han SW Kim HJ Varying Dialysate Bicarbonate Concentrations in Maintenance Hemodialysis Patients Affect Post-dialysis Alkalosis but not Pre-dialysis Acidosis Electrolyte & Blood Pressure 2007 5 2 95 101 10.5049/EBP.2007.5.2.95 24459507 PMC3894521

[B25] Fan S Ross C Mitra S Kalra P Heaton J Hunter J Plone M Pritchard N A randomized, crossover design study of sevelamer carbonate powder and sevelamer hydrochloride tablets in chronic kidney disease patients on haemodialysis Nephrol Dial Transplant 2009 24 12 3794 10.1093/ndt/gfp372 19666658 PMC2781155

[B26] Hutchison AJ Laville M Switching to lanthanum carbonate monotherapy provides effective phosphate control with a low tablet burden Nephrol Dial Transplant 2008 23 11 3677 10.1093/ndt/gfn310 18577536 PMC2568007

[B27] Sprague S Ross E Nath S Zhang P Pratt R Krause R Lanthanum carbonate vs. sevelamer hydrochloride for the reduction of serum phosphorus in hemodialysis patients: a crossover study Clin Nephrol 2009 72 4 252 258 19825330 10.5414/cnp72252

[B28] Filiopoulos V Koutis I Trompouki S Hadjiyannakos D Lazarou D Vlassopoulos D Lanthanum Carbonate Versus Sevelamer Hydrochloride: Improvement of Metabolic Acidosis and Hyperkalemia in Hemodialysis Patients Ther Apher Dial 2011 15 1 20 27 10.1111/j.1744-9987.2010.00868.x 21272248

